# High Subsidence Rate After Primary Total Hip Arthroplasty Using a Zweymüller-type Noncemented Implant With a Matte Surface

**DOI:** 10.5435/JAAOSGlobal-D-21-00126

**Published:** 2022-06-07

**Authors:** Toshiyuki Kawai, Koji Goto, Yutaka Kuroda, Yaichiro Okuzu, Shuichi Matsuda

**Affiliations:** From the Department of Orthopedic Surgery, Kyoto University Graduate School of Medicine, Sakyo-ku, Kyoto, Japan.

## Abstract

**Introduction:**

The surface topography is one key factor that affects the initial fixation of prosthesis in total hip arthroplasty (THA). We aimed to evaluate the mid-term results of a Zweymüller-type noncemented femoral implant (Elance stem) that had a matte surface with a target average roughness of 1.0 to 2.5 μm. The prosthesis was subjected to alkali and heat treatments to enhance its bone-bonding property.

**Methods:**

In this retrospective study, 30 THAs (27 patients) done using an Elance stem from September 2012 to October 2014 were evaluated clinically and radiographically for a mean follow-up of 6.3 ± 1.7 years after the index THA.

**Results:**

Stem revision was indicated for six hips (20%). The survival rate with stem revision for any reason was 86.4% (95% confidence interval, 68.9%-94.8%) at 5 years. Stem subsidence >5 mm was noted in 17 hips (56.7%). The survival rate with stem subsidence >5 mm as the end point was 46.6% (95% confidence interval, 29.9%-64.2%) at 5 years.

**Conclusion:**

The Zweymüller-type noncemented stem with a low-roughness matte surface demonstrated a high subsidence rate, although the bone-bonding property was potentially enhanced by the alkali and heat treatments. Surgeons should be aware that an insufficient surface roughness could lead to poor mechanical fixation of the noncemented stem, even with an appropriate stem geometry and surface chemistry.

Total hip arthroplasty (THA) is a common surgical procedure that improves the quality of life of patients with end-stage arthritis by decreasing pain and improving motor function and mobility as measured by validated health-related outcome tools.^1,2^

Various noncemented femoral stems have been associated with excellent long-term clinical and radiographic outcomes.^3^ Initial fixation is obtained by press-fitting a slightly oversized implant. A number of factors influence the initial stability or primary fixation. These include geometry, roughness, and coating of the stem; technique of preparation; and bone quality.^3^ Several methods have been reported to enhance the bioactivity of metal implants. One of the best accepted and commercialized bioactive coating materials is plasma-sprayed hydroxyapatite (HA).^4,5^ However, a large retrospective study from the Scandinavian Joint Registry demonstrated that the HA and non–HA-coated prostheses have been shown to have similar revision rates.^6^

Alkaline and heat treatments introduced by Kokubo et al^7^ as a surface modification of titanium (Ti) and its alloys provided them with bioactivity. Ti metal that had been soaked in a NaOH solution and then heat-treated possessed apatite-forming ability on its surface in simulated body fluid. Several animal studies have demonstrated the enhanced bone-bonding ability of Ti metals after alkali and heat treatments.^8,9^ Alkali-heat treatment applied on a thermally sprayed, rough Ti metal surface was reported to confer higher bone-bonding properties than a HA-coated, thermally sprayed, rough Ti metal surface, as demonstrated in a rabbit model.^10^ A noncemented metaphyseal fit-type femoral implant with plasma-sprayed Ti metal on its proximal body that had been subjected to alkaline and heat treatments has been developed and used in clinical practice since 2007. The reported implant survival was 100% at 5 years and 98% at 10 years with no stem loosening.^11,12^ Subsequently, a Zweymüller-type noncemented implant with a sandblasted surface that had been subjected to alkali and heat treatments was introduced in Japan in 2012. It has a matte surface with a relatively low average roughness of 1.0 to 2.5 μm, whereas other Zweymüller-type stems have a larger average target roughness of 4.0 to 8.0 μm.

The goal of this study was to clinically and radiographically evaluate mid-term results of the alkali-treated and heat-treated Zweymüller-type stem that had a matte surface with a relatively low roughness.

## Methods

This retrospective study included patients who underwent primary THA using an Elance femoral implant at Kyoto University Hospital. All patients provided informed consent, and the study protocol was approved by the institutional review board of our hospital.

From September 2012 to October 2014, a primary THA was done for 217 hips at our institute, of which 30 hips (27 patients) received the Elance femoral implant. The indication for surgery was osteoarthritis (OA) secondary to hip dysplasia in 23 hips, osteonecrosis of the femoral head in 5 hips, primary OA in 1 hip, and rheumatoid arthritis in 1 hip. The choice of implant was according to surgeon's preference.

**Figure 1:**
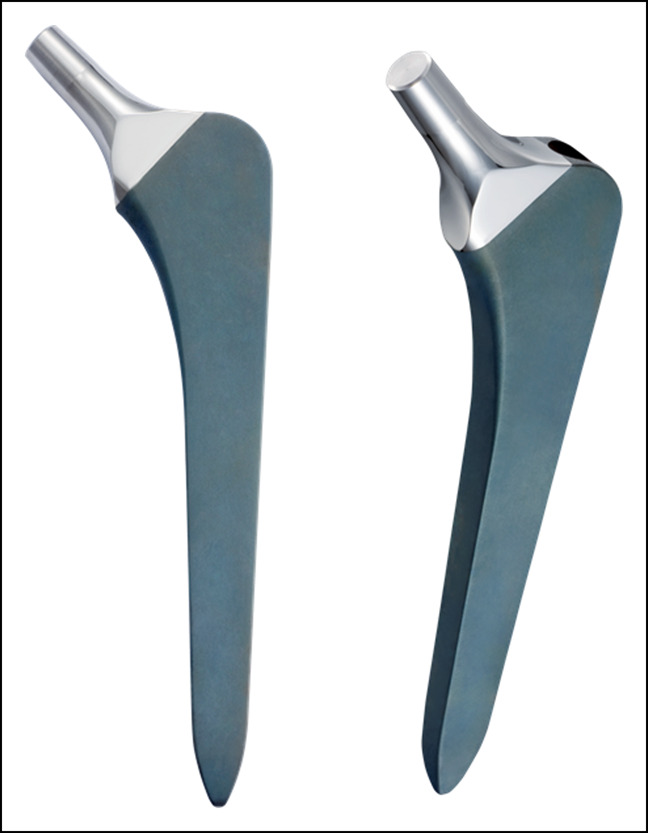
Photographs of the Elance femoral stem.

The Elance stem (Kyocera) (Figure 1) is a type of Zweymüller-type noncemented femoral stem that is straight-tapered at both the medial-lateral and AP planes (rectangular cross-section) without any trochanteric shoulder. To obtain a matte surface texture, surface sandblasting was done on the whole stem using an abrasive paper with a target roughness of 1.0 to 2.5 μm. After sandblasting, alkali and heat treatments were done using a previously reported procedure.^8,9^ Briefly, the stem was soaked in a 5.0 M NaOH aqueous solution at 60°C for 24 hours and then gently washed with distilled water and dried at 40°C for 24 hours at room temperature. The stem was subsequently heated to 600°C at a rate of 5°C/min in an electric furnace and held for 1 hour before cooling to room temperature.

The acetabular implant used was a noncemented cup (AHFIX Q3 cup; Kyocera) with a highly cross-linked polyethylene (Aquala liner; Kyocera) in all hips. The femoral head used was a zirconia-toughened alumina ball (AZ209; Kyocera) in all hips.

Three experienced senior surgeons, who each had more than 15 years of experience, conducted all surgical procedures in accordance with the manufacturer's instructions by an anterolateral approach. Femoral reaming was done in a standardized manner with the use of a pneumatic broaching system (Woodpecker, Integrated Medical Technologies USA, LLC). Full weight bearing was allowed immediately after surgery.

### Clinical and Radiographic Evaluation

Patients were evaluated clinically and radiographically at 3, 6, and 12 months after surgery and annually thereafter. Hip function was evaluated by two authors (Y.K. and K.S.; one of them was involved in the surgeries) according to the Japan Orthopaedic Association Hip Score (JOA score).^13^

One author (T.K.; not involved in the surgeries) examined all radiographs for osteolytic lesions (>2 mm in width and progressive),^14^ reactive lines (radio-opaque lines adjacent to the implants),^15^ stress shielding,^16^ pedestal formation,^15^ and cortical hypertrophy^17^ from AP radiographs obtained after surgery. Stress shielding was assessed using the criteria of Engh et al.^16,18^ The locations of the reactive lines were recorded according to the Gruen zones for the proximal femur.^19^ For measuring stem subsidence, the distance between the most proximal point of the greater trochanter and the shoulder of the femoral stem on AP radiographs was measured at each postoperative year compared with the radiograph immediately after surgery. The measurements were calibrated according to the known head size of the femoral prosthesis.

We observed the surface of one Elance stem retrieved during revision THA conducted for stem subsidence at 8 years after primary THA in this cohort to assess any structural changes in the stem surface and residual bone on the stem surface. The stem was immersed in 10% phosphate-buffered formaldehyde for 3 days and dehydrated in serial concentrations of ethanol (5%, 70%, 80%, 90%, 99%, 100%, and 100% vol/vol) for 3 days in each concentration. This is a standard procedure for observing the surface of an explanted device and the interface between implant and bone tissue. Surface observation was done using a scanning electron microscope (SEM; S-3400 N, Hitachi) attached to an energy-dispersive radiograph microanalyzer (Genesis; Ametek).

### Statistical Analysis

The comparison of JOA scores before surgery and at the final follow-up and the comparison of JOA scores at the final follow-up between patients with subsidence >5 mm and other patients were done using a Student *t*-test, with *P* < 0.05 being statistically significant. The JOA scores at the final follow-up for patients who underwent revision surgery were excluded from those analyses. Differences in proportions were calculated using the Pearson chi square test.

The survival rate was measured with stem revision for any reason as an end point. The other 187 THAs done using a stem other than Elance comprised the control group for the comparison of stem survivorship with the Elance group and to examine the potential selection bias toward the use of the Elance stem. The demographic characteristics and type of stem used are summarized in Table 1. A log-rank regression analysis was conducted to compare the survivorship between the Elance and control groups, with stem revision for any reason as an end point. A separate survival rate was also calculated with stem subsidence >5 mm as an end point. The cumulative subsidence was calculated at each time point. For cases with subsequent revision, the subsidence data until revision surgery were included in this analysis. All statistical analyses were conducted using JMP Pro 14 software (SAS Institute).

**Table 1:** Patients' Demographics

Variable	Elance Users (27 Patients, 30 Hips)	Other Stem (174 Patients, 187 Hips)	*P* Value
Age	60.3 ± 14.3 (23-82)	64.6 ± 10.9 (30-86)	0.14
Sex (female)	23/27 (85.2)	143/174 (82.2)	0.70
BMI (kg/m^2^)	23.0 ± 3.4 (18.2-31.3)	23.2 ± 3.9 (15.6-36.0)	0.85
Indication			
OA secondary to DDH	23/30 (76.7)	142/187 (75.9)	0.68
ONFH	5/30 (16.7)	30/187 (16.0)	
Primary OA	1/30 (3.3)	13/187 (7.0)	
Rheumatoid arthritis	1/30 (3.3)	2/187 (1.1)	
Stem used			
Noncemented	Elance (Kyocera): 30	SL-PLUS (Smith & Nephew): 72	
		J-Taper (Kyocera): 31	
		ANTHOLOGY (Smith & Nephew): 4	
		S-ROM (DePuy Synthes): 4	
		H10 (Kyocera): 1	
Cemented		KMAX SS TAPER (Kyocera): 72	
		Type 6 (Kyocera): 3	

BMI = body mass index, DDH = developmental dysplasia of the hip, OA = osteoarthritis, ONFH = osteonecrosis of the femoral head

Data are shown as average ± SD (range) or n (%).

## Results

Demographic data are summarized in Table 1. For the Elance group, the mean clinical follow-up period was 6.3 ± 1.7 years, whereas the mean follow-up period for radiograph analysis was 6.2 ± 1.7 years. Although the average age of the Elance group was slightly younger than that of the control group (patients undergoing THA with stems other than the Elance stem), the difference was not significant (60.3 ± 14.3 vs 64.6 ± 10.9 years, *P* = 0.14). When the Elance group was compared with the group of patients who underwent THA with other noncemented stems, the difference in the age was small (60.3 ± 14.3 vs 61.3 ± 10.4 years, *P* = 0.68). The JOA score improved from preoperative 49.9 ± 15.5 to 82.5 ± 10.1 at the final follow-up (*P* = 0.019) (Table 2).

**Table 2:** JOA Score for Elance Users Before Surgery and at the Last Follow-up

Variable	Preoperative JOA Score	JOA Score at the Final Follow-up	*P* Value
Pain	14.3 ± 8.0 (0-30)	35.4 ± 4.5 (10-40)	<0.001
Range of motion	12.2 ± 3.9 (4-19)	16.1 ± 3.0 (7-20)	<0.001
Walk	10.1 ± 4.0 (0-18)	14.5 ± 3.5 (10-20)	<0.001
Activity of daily living	13.2 ± 3.9 (2-20)	16.2 ± 2.4 (12-20)	0.0015
Total	49.9 ± 15.5 (6-80)	82.5 ± 10.1 (71-100)	<0.001

JOA = Japan Orthopaedic Association

Data are shown as average ± SD (range).

**Figure 2:**
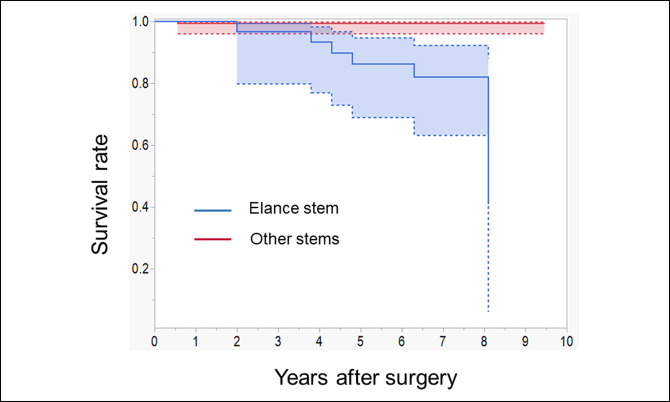
Graph showing Kaplan-Meier survivorship analysis with revision for any reason as the end point. The dashed lines indicate 95% confidence interval.

No infections, dislocations, or periprosthetic fractures were reported in the Elance group. Stem revision was indicated for 6 hips (20%). The survival rate in the Elance group with stem revision for any reason was 86.4% (95% confidence interval [CI], 68.9%-94.8%) at 5 years (Figure 2); in comparison, the control group (THA done using stems other than the Elance stem) had a significantly higher survival rate of 99.5% (95% CI, 96.2%-99.9%). Only one patient in the control group underwent revision for septic loosening at 6 months after primary hybrid THA.

**Figure 3:**
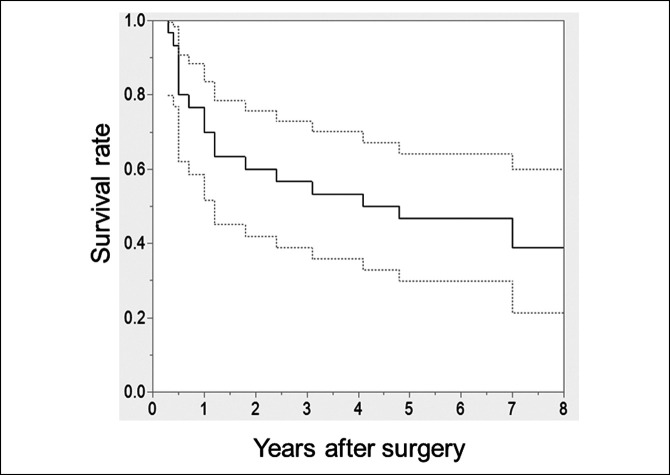
Graph showing Kaplan-Meier survivorship analysis with subsidence >5 mm as the end point. The dashed lines indicate 95% confidence interval.

**Figure 4:**
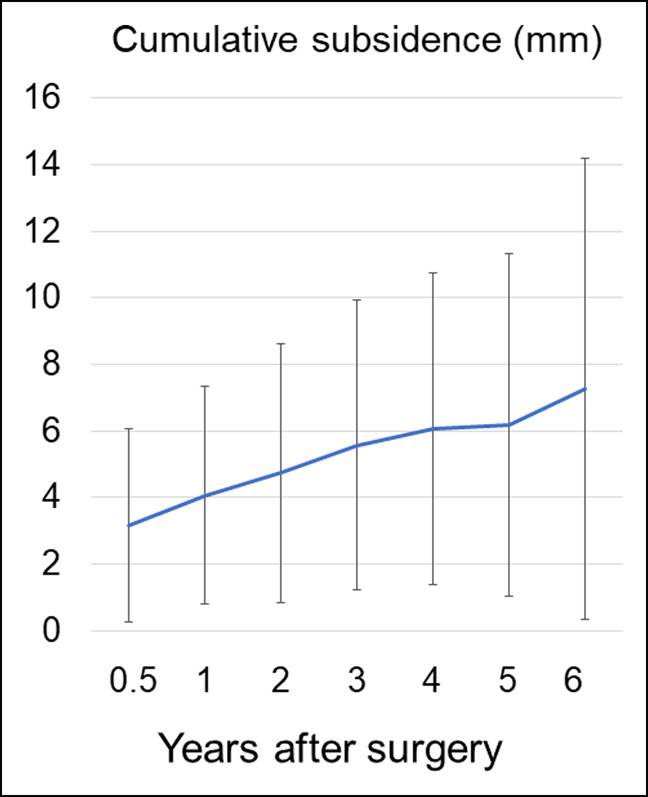
Graph showing cumulative subsidence after surgery.

**Figure 5:**
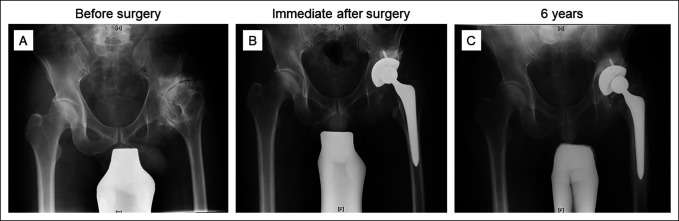
**A**, Radiographs showing a 58-year-old man presented with left hip pain owing to end-stage osteoarthritis secondary to dysplasia. **B**, Primary total hip arthroplasty using an Elance stem was done. **C**, The stem subsided by 26.4 mm at 6 years after surgery, which resulted in a shortened limb. He was scheduled for revision surgery, but the schedule is being readjusted owing to the COVID-19 pandemic. He is still on a waitlist.

Stem subsidence >5 mm was noted in 17 hips (56.7%) in the Elance group. The incidence of stem subsidence >5 mm in accordance with the diagnosis was 15 of 23 (65.2%) for patients with OA secondary to developmental dysplasia of the hip (DDH), 1 of 1 (100%) for those with primary OA, and 1 of 5 (20%) for those with osteonecrosis of the femoral head. The survival rate with stem subsidence >5 mm as the end point was 46.6% (95% CI, 29.9%-64.2%) at 5 years (Figure 3). The average cumulative stem subsidence was 4.1 ± 3.3 mm at 1 year after surgery and then gradually increased (Figure 4). Six stems in the Elance group required revision at 2.0, 3.8, 4.3, 4.8, 6.3, and 8.1 years, respectively, after index primary THA. The indication for stem revision was severe thigh pain in 6 hips with stem subsidence >5 mm. Although the stem was revised with a cemented stem in 5 hips, the subsided stem was kept in place in the remaining case because the patient could not visit our institute to undergo revision surgery owing to the COVID-19 pandemic. He was unable to walk owing to pain, and the date of revision surgery is under readjustment. The features of this case are presented in Figure 5. The case was considered as under revision in the survival rate analysis at the time revision surgery was scheduled. In the other five revision cases, the thigh pain was completely resolved after the revision with a cemented stem.

The radiographic findings in the Elance group are summarized in Table 3. Reactive lines were observed in 25 hips (83.3%), whereas stress shielding was noted in 23 hips (76.7%). The comparisons of incidences of a reactive line (any zone), stress shielding, osteolysis, pedestal formation, and cortical hypertrophy between patients with subsidence >5 mm and other patients are summarized in Table 4. Pedestal formation was more frequently seen in patients with a subsidence of >5 mm (*P* = 0.0042). The comparison of JOA scores between patients with subsidence >5 mm and other patients is presented in Table 5. Significant differences were observed between the two groups in pain component, walking component, and total score (*P* = 0.0059, 0.016, and 0.0016, respectively), whereas no significant difference was observed in the range-of-motion component (*P* = 0.12). The activities-of-daily-living component score tended to be lower in the subsidence group, but the difference was not statistically significant (*P* = 0.051).

**Table 3:** Radiographic Findings of 30 Hips at the Mean 6.2-Year Follow-up

Finding	N (%)
Reactive line	
Zone 1	24 (80.0)
Zone 2	15 (50.0)
Zone 3	7 (23.3)
Zone 4	5 (16.7)
Zone 5	9 (30.0)
Zone 6	15 (50.0)
Zone 7	23 (76.7)
No reactive line	5 (16.7)
Stress shielding	
Grade 1	11 (36.7)
Grade 2	10 (33.3)
Grade 3	2 (6.7)
Grade 4	0 (0)
Pedestal formation	20 (66.7)
Osteolysis	0 (0)
Cortical hypertrophy	10 (33.3)
Subsidence >5 mm	17 (56.7)

Stress shielding grade 1: Only the most proximal medial edge of the cut femoral neck was rounded off slightly; grade 2: Rounding off of the proximal medial femoral neck was combined with loss of medial cortical density at stem zone 7; grade 3: more extensive resorption of the cortical bone involving the medial cortex at stem zones 6 and 7; grade 4: cortical resorption extended below zones 6 and 7 into the diaphysis.^17^

**Table 4:** Comparison of the Incidence of Radiographic Findings Between Patients With Stem Subsidence >5 mm and Other Patients (for Elance Users)

Finding	No Subsidence (n = 13)	Subsidence (n = 17)	*P* Value
Reactive line	9 (69.2)	16 (94.1)	0.070
Stress shielding	9 (69.2)	14 (82.4)	0.40
Osteolysis	0	0	1
Pedestal formation	5 (38.5)	15 (88.2)	0.0042
Cortical hypertrophy	4 (30.8)	6 (35.3)	0.79

**Table 5:** Comparison of JOA Scores for Elance Users at the Last Follow-up Between Patients With Stem Subsidence >5 mm and Other Patients

Variable	No Subsidence (n = 13)	Subsidence (n = 11)	*P* Value
Pain	37.7 ± 3.9 (30-40)	32.7 ± 4.1 (30-40)	0.0059
Range of motion	17.1 ± 2.8 (9-20)	15.1 ± 3.2 (10-19)	0.12
Walk	16.2 ± 3.6 (10-20)	12.7 ± 2.6 (10-15)	0.016
Activity of daily living	17.2 ± 2.9 (12-20)	15.3 ± 1.3 (12-16)	0.051
Total	88.2 ± 9.8 (73-100)	75.8 ± 6.3 (71-90)	0.0016

JOA = Japan Orthopaedic Association

The six cases indicated for revision surgery were excluded from this analysis. Data are shown as average ± SD (range).

**Figure 6:**
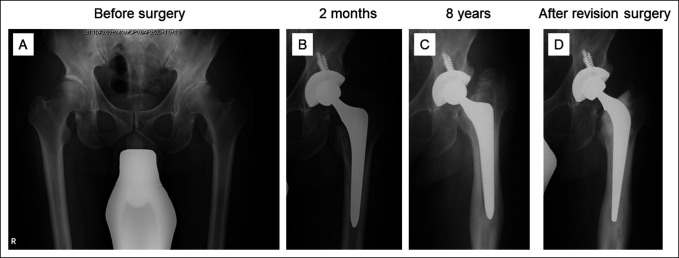
**A**, Radiographs showing a 57-year-old man presented with right hip pain caused by severe osteoarthritis secondary to dysplasia. **B**, He underwent primary total hip arthroplasty using an Elance stem. **C**, Radiograph obtained at 8 years after surgery. A massive stem subsidence of 27.8 mm was observed. **D**, The patient underwent an isolated stem revision using a cemented stem. The stem was manually mobilized and was easily removed from the femoral canal with a hammer. No signs of infection such as abnormal fluid collection were observed during the surgery.

**Figure 7:**
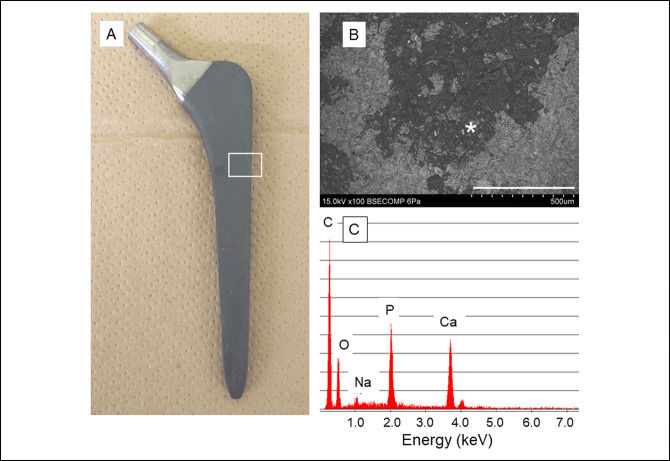
Analysis of the surface of a retrieved stem. **A**, Photograph of the stem removed owing to massive subsidence. No bone tissue was observed on the surface under macroscopic observation. **B**, SEM image at the area indicated by a white box in **A** (scale bar, 500 μm). Small fragments were attached on the surface of the stem. **C**, SEM energy-dispersive radiograph analysis of the area indicated by an asterisk in **B** revealing calcium and phosphorus in the fragments, suggesting that the fragments were small pieces of bone tissue.

The representative failure cases are shown in Figures 5 and 6. Almost no bone tissue was observed on the surface of the stem removed in the case in Figure 6 by macroscopic inspection (Figure 7, A). SEM examinations revealed thin small tissues on the surface (Figure 7, B). Energy-dispersive radiograph analysis revealed calcium and phosphate peaks (Figure 7, C), implying that the small pieces observed on the surface were thin bone fragments. However, the area containing those bone-like tissues was very small, indicating that bone bonding between the stem and the femoral bone was limited.

## Discussion

In this study, stress shielding was observed in 76.7% in total and 43.3% for Grade 2 or higher when the Elance stem was used. Zweymüller-type stems are designed for a diaphyseal press-fit fixation that leads to proximal stress shielding and diaphyseal cortical hypertrophy. Stress shielding was observed in 51% to 60% in previous studies.^20,21^

Cortical hypertrophy after implantation of Zweymüller-type stems was seen in 42.0% to 69.9% of the cases in previous studies,^21,22^ whereas it was observed in 33.3% in this study. These findings are attributed to the concentration of stress in the transitional zone between the stiff stem and the elastic cortical bone. The incidence of stress shielding observed in this study was comparable with that reported in previous studies, although the stress-shielding rate was relatively higher in hips with subsidence >5 mm. We think that a high subsidence rate might have led to a smaller mechanical loading on the affected limb, resulting in a higher stress-shielding rate.

Stem subsidence is considered a relevant factor for early failure of THA. The risk of femoral stem subsidence before osseointegration is reported with rates of 5% to 61.5% in elective hip arthroplasty surgery.^23,24^ For many authors, >5 mm of subsidence is a negative factor suggesting implant instability. Conversely, if no subsidence occurs, satisfactory osseointegration and long-term stability should be guaranteed.^15,25^

The subsidence and revision rates in this study were much higher than those reported for the AHFIX stem, which is a previously released noncemented, plasma-sprayed Ti metal structure, alkali-heat–treated stem. The short-term and long-term follow-up results of THA using the AHFIX stem at our institute have previously been reported.^11,12^ Using revision for any reason as the end point, the overall survival rate of the AHFIX stem was 98% (95% CI, 96%-100%) at 10 years. No implant had radiographic signs of loosening.^12^

For the AHFIX stem, 4 hips (6%) had initial subsidence (3 mm in 3 hips and 5 mm in 1 hip) within 3 months after operation, but no progressive subsidence was observed after this period.^12^ The main differences between the two stems subjected to both alkali and heat treatments were stem geometry and surface topography. Although the AHFIX stem had a plasma-sprayed porous structure on its proximal part with a thickness of 700 μm, the entire Elance stem had a sandblasted matte surface with a target Ra of 1.0 to 2.5 μm. The plasma-sprayed surface of the AHFIX stem may have contributed to a good initial fixation, which resulted in a low subsidence (>5 mm) rate (1.4%) compared with that of the Elance stem (56.7%) in this study. Although the prevalence of DDH was relatively high (76.7%) in this study, this is unlikely to be the reason for the high failure rate in this cohort because the previous study that showed a much lower subsidence rate for another alkali-heated stem also had a high prevalence of DDH (94.0%).^12^

The Zweymüller-type stem is a rectangular-tapered stem that is sandblasted across its entire length. It has a rectangular cross-section that provides a three-point fixation at the metaphyseal-diaphyseal junction and proximal part of the diaphysis. Its cross-section enables a four-point, antirotational stability.^26^ Excellent long-term stem survivorships have been reported for Zweymüller-type stems (with stem revision for any reason as the end point): 98% at 15 years,^27^ 98% at 17 years,^28^ 95% at 18 years,^29^ and 84.7% at 30 years.^30^ The Elance stem had a smaller lateral shoulder than the original Zweymüller stem; however, the SL-PLUS MIA stem (Smith & Nephew), which had an even smaller lateral shoulder than the original Zweymüller, showed a 100% stem survival rate at 6 years and had an average subsidence of 0.5 mm.^31^ Based on these findings, the high failure rate of the Elance stem cannot be attributed to its stem geometry.

The Elance stem had a sandblasted matte surface with a target Ra of 1.0 to 2.5 μm, whereas the other Zweymüller stems had a higher roughness with a target Ra of 4 to 8 μm for SL-PLUS (Smith & Nephew) and Alloclassic (Zimmer-Biomet). This difference in surface roughness may have affected the initial fixation of the stem. A previous report described that an implant with a rough surface has a favorable effect on early and strong bonding to the bone.^32,33^ SL-PLUS implants were evaluated for their femoral implant using the Ein Bild Roentgen Analysis in a previous study. Mean migration was 0.3 ± 0.8 mm at 2 years.^31^ The survival rate for the Alloclassic stem with subsidence >5 mm as the end point was 88.5% at 12 years.^20^ Conversely, we found that the survival rate of the Elance stem with subsidence >5 mm as the end point was 56.7% at 5 years with a mean subsidence of 4.9 mm at 2 years. In this study, the cases with subsidence >5 mm were more likely to suffer pain and have lower walking ability (Table 2). A subsidence of >5 mm was associated with a poorer clinical outcome. Furthermore, subsidence >5 mm was associated with pedestal formation (*P* = 0.0042). Distal pedestal formation implies implant-to-bone stress transfer away from the metaphyseal part of the implant and is associated with instability.^34^ The formation of this shelf of a new bone is considered an attempt to support the tip of the unstable prosthesis.

The effects of the alkali and heat treatments on the bone-bonding properties of Ti metals and its alloys have been examined in previous studies.^8,9^ However, the stem used in this study, which had been subjected to alkali and heat treatments, demonstrated relatively poor implant fixation. Hackling et al suggested that the contribution of surface topography is much larger than that of surface chemistry.^35^ The high failure rate of initial fixation in the stem used in this study can be attributed to the insufficient surface roughness. After the potential tendency to subside was noted in late 2014, the Elance stem was no longer used at our institute.

This study has several limitations. First, this study had a small sample size and a retrospective design. The number was small (30 hips) in the Elance group because we stopped using this stem soon after high subsidence was noted. However, we thought that the difference in the subsidence rate between this cohort and previous studies was so large that the data presented in this study can be sufficiently conclusive. Second, the cohort included patients with various indications for THA. Third, patient selection bias may have occurred because implant selection was at the surgeons' preference, which could have affected the results. Fourth, this study lacked a control group. Because the survivorships and subsidence rates for other Zweymüller stems have been rigorously reported, comparison with the literature could highlight the high failure rate of the stem used in this cohort.

In conclusion, the Zweymüller-type noncemented stem with a low-roughness matte surface demonstrated a high subsidence rate, although the bone-bonding property was potentially enhanced by the alkali and heat treatments. The insufficient surface roughness could have led to poor mechanical fixation of the noncemented stem, even when the stem had an appropriate geometry and surface chemistry.
